# Associations of alternative cannabis product use and poly-use with subsequent illicit drug use initiation during adolescence

**DOI:** 10.1007/s00213-023-06330-w

**Published:** 2023-03-03

**Authors:** Jessica L. Braymiller, Kira E. Riehm, Madeline Meier, Evan A. Krueger, Jennifer B. Unger, Jessica L. Barrington-Trimis, Junhan Cho, H. Isabella Lanza, Danielle R. Madden, Afton Kechter, Adam M. Leventhal

**Affiliations:** 1 Department of Population and Public Health Sciences, University of Southern California, Keck School of Medicine, Los Angeles, CA 90033, USA; 2 Department of Mental Health, Johns Hopkins University, Bloomberg School of Public Health, Baltimore, MD 21205, USA; 3 Department of Psychology, Arizona State University, Tempe, AZ 85281, USA; 4 Institute for Addiction Science, University of Southern California, 2001 N Soto Street, #302-C, Los Angeles, CA 90032, USA; 5 USC Norris Comprehensive Cancer Center, Los Angeles, CA 90033, USA; 6 Department of Human Development, California State University, Long Beach, CA 90840, USA; 7 USC Suzanne Dworak-Peck School of Social Work, University of Southern California, Los Angeles, CA 90089, USA; 8 Department of Psychology, University of Southern California, Los Angeles, CA 90033, USA

**Keywords:** Cannabis, Vaping, Edible, Adolescents, Poly-substance use

## Abstract

**Rationale:**

Specific cannabis products may differentially increase risk of initiating non-cannabis illicit drug use during adolescence.

**Objective:**

To determine whether ever- and poly-use of smoked, vaporized, edible, concentrate, or blunt cannabis products are associated with subsequent initiation of non-cannabis illicit drug use.

**Methods:**

High school students from Los Angeles completed in-classroom surveys. The analytic sample (*N* = 2163; 53.9% female; 43.5% Hispanic/Latino; baseline *M* age = 17.1 years) included students who reported never using illicit drugs at baseline (spring, 11th grade) and provided data at follow-up (fall and spring, 12th grade). Logistic regression models assessed associations between use of smoked, vaporized, edible, concentrate, and blunt cannabis at baseline (yes/no for each product) and any non-cannabis illicit drug use initiation—including cocaine, methamphetamine, psychedelics, ecstasy, heroin, prescription opioids, or benzodiazepines—at follow-up.

**Results:**

Among those who never used non-cannabis illicit drugs at baseline, ever cannabis use varied by cannabis product (smoked = 25.8%, edible = 17.5%, vaporized = 8.4%, concentrates = 3.9%, and blunts = 18.2%) and patterns of use (single product use = 8.2% and poly-product use = 21.8%). After adjustment for baseline covariates, odds of illicit drug use at follow-up were largest for baseline ever users of concentrates (aOR [95% CI] = 5.74[3.16–10.43]), followed by vaporized (aOR [95% CI] = 3.11 [2.41–4.01]), edibles (aOR [95% CI] = 3.43 [2.32–5.08]), blunts (aOR [95% CI] = 2.66[1.60–4.41]), and smoked (aOR [95% CI] = 2.57 [1.64–4.02]) cannabis. Ever use of a single product (aOR [95% CI] = 2.34 [1.26–4.34]) or 2 + products (aOR [95% CI] = 3.82 [2.73–5.35]) were also associated with greater odds of illicit drug initiation.

**Conclusions:**

For each of five different cannabis products, cannabis use was associated with greater odds of subsequent illicit drug use initiation, especially for cannabis concentrate and poly-product use.

## Introduction

Smoking cannabis flower has long been the most prevalent form of cannabis use. However, recent changes in cannabis legalization and commercialization have increased the availability of alternative cannabis products that may appeal to youth, including edible products (i.e., cannabinoid-infused food and drinks), vaporized cannabis (i.e., electronic vaporizers used to heat cannabis flower or liquid extracts into inhalable aerosol with minimal or no combustion), cannabis concentrates (i.e., extracts with high concentrations of tetrahydrocannabinol [THC]), and blunts (i.e., cannabis flower rolled in tobacco cigar casing; (Spindle et al. 2019). The diversity of cannabis products available also provides opportunity for poly-cannabis use (i.e., use of ≥ 2 cannabis products; (Peters et al. 2018; Schauer et al. 2020). While adolescent cannabis use and poly-use of various cannabis products is fairly prevalent (Schauer et al. 2020), associations with adverse illicit drug use outcomes are largely uninvestigated.

Previous longitudinal studies have found an association of cannabis use with subsequent initiation of non-cannabis illicit drug use (Noel and Wang 2018), which in turn may increase odds of numerous adverse consequences in adulthood, such as incarceration (Slade et al. 2008), increased job instability (Bentler 1992), the development of substance use disorders (Chen et al. 2009; King and Chassin 2007), and psychiatric disorders (Brook et al. 2000), among others. However, existing studies on this topic were conducted prior to the raising popularity of alternative cannabis products and focused on exposure to only smoked flower cannabis. The extent to which the association of cannabis use with subsequent illicit drug use initiation extends to various types of cannabis products use and poly-cannabis use is unknown.

Certain characteristics of alternative cannabis products (e.g., availability of flavorings, absence of odor, and lack of airway irritation from smoke) might draw in adolescents who otherwise would not have used cannabis (Friese et al. 2016; Kenne et al. 2017). Furthermore, cannabis products that deliver higher doses of THC (e.g., concentrates) could generate more rapid, pleasurable psychoactive effects (Ewusi Boisvert et al. 2020; Spindle et al. 2019). As such, increased risk of illicit drug use initiation could be particularly robust for certain cannabis products. The current study investigated the association of use and poly-use of 5 different cannabis products (smoked, vaporized, edible, concentrates, or blunts) with subsequent non-cannabis illicit drug use initiation in adolescents in Southern California, the US state with the highest prevalence of alternative cannabis product use (Schauer et al. 2020).

## Methods

### Participants and procedures

Data were from a prospective cohort study including 10 Los Angeles area high schools. Students completed surveys once every 6 months beginning of fall 2013 (9th grade; (Kelley-Quon et al. 2019). Students absent for data collections, who moved, or who dropped out of schools completed phone/mail/web surveys. The use of each of the alternative cannabis products of interest in the current study was first assessed in the spring 2016 11th grade survey (baseline for this study). Twelve-month follow-up included illicit drug use data collected in the fall and spring 12th grade surveys (2016–2017). The University of Southern California Institutional Review Board approved this study. Participating students provided parental consent and student assent.

**Fig. 1:**
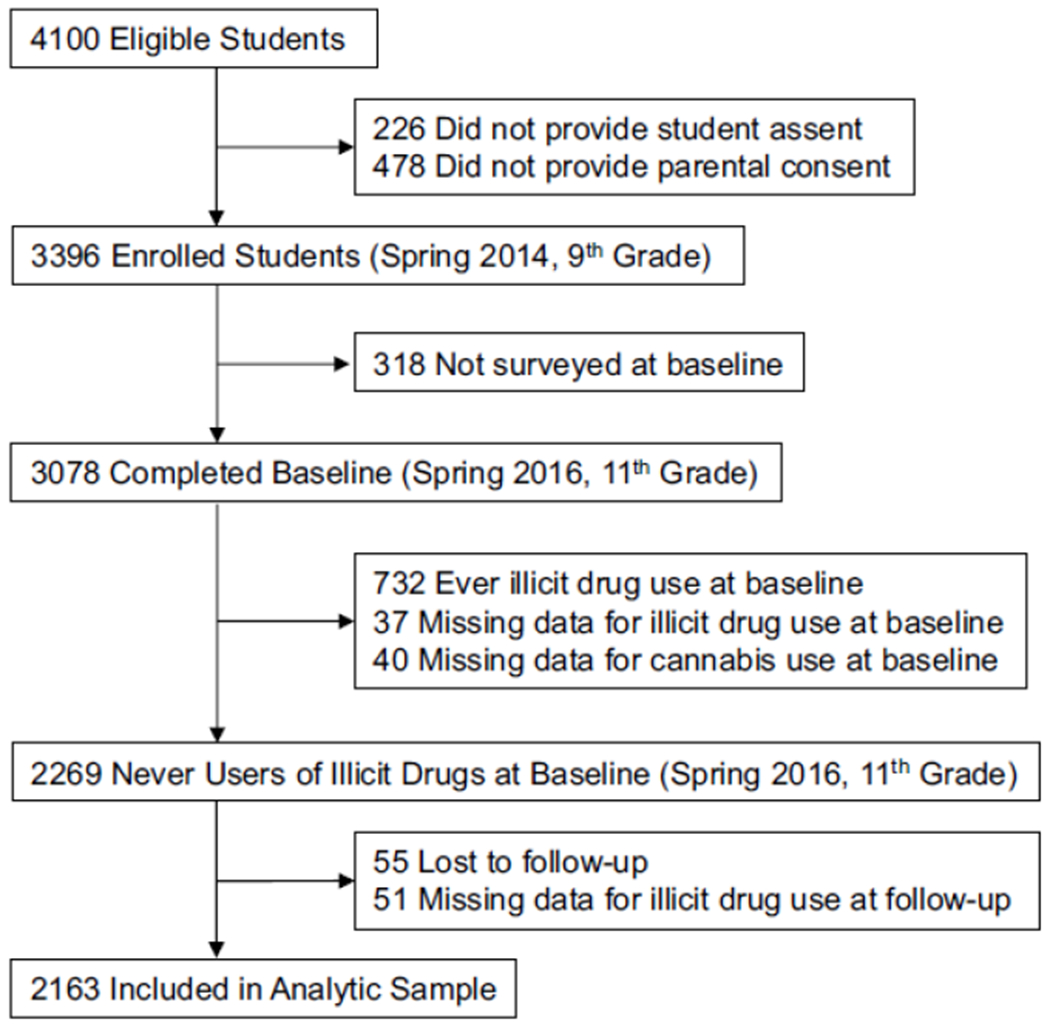
Flow diagram for inclusion in study analytic sample

Of students enrolled in the participating schools in fall 9th grade (*n* = 4100), 3396 (83%) provided assent and parental consent. Among students with baseline (spring 11th grade; 2016) cannabis and non-cannabis illicit drug use data (*N* = 3001), 732 (24.4%) ever users of illicit drugs at baseline were excluded from analyses (number of observations available for each study covariate in analytic sample reported in eTable 1; frequencies for specific illicit drugs reported in eTable 2). Among baseline never users of non-cannabis illicit drugs, 2163 (95.3%) provided illicit drug use data over the 12-month follow-up, constituting this study’s analytic sample (Fig. 1). Those excluded (vs. included) in the analytic sample differed in racial/ethnic composition were less likely to have parents with a college degree, were slightly older, and more likely to be male, and ever used alcohol, tobacco products, or smoked cannabis flower by fall 9th grade (see eTable 3).

### Measures

#### Baseline cannabis product use

At baseline, participants completed 5 self-report items adapted from national epidemiologic surveillance surveys^10^ that assessed ever use of combustible flower cannabis (item wording: “smoked marijuana [pot, weed, hash, reefer, bud, or grass]”); edible cannabis (“marijuana or THC food or drinks [pot brownies, cookies, cakes, butter, oil]”); vaporized cannabis (“electronic device to vape marijuana or hash oil [liquid pot, weed pen]”); concentrates (“dabbing [wax, shatter, budder, butane hash oil, BHO]”); and blunts (“blunts [marijuana rolled in tobacco leaf or cigar casing]”). Blunts were assessed separately from other methods of cannabis use due to co-administration of cannabis and nicotine, as well as the popularity and attractiveness of blunts among young people (Antognoli et al. 2018; Delnevo et al. 2011; Trapl et al. 2018). Individual responses for each item (yes/no) served as five primary exposure variables. The total number of cannabis products ever used (range 0–5, recoded trichotomously due to small cell counts of individuals who used 3, 4, or 5 cannabis products: 0 vs. 1 vs. ≥ 2) was an additional primary exposure variable reflecting poly-cannabis use. Parallel past 30-day cannabis product use measures were also collected and reported for descriptive purposes but not analyzed as exposure variables due to low cell counts.

#### Follow-up illicit drug use initiation

In the fall and spring of 12th grade (follow-up assessments), 7 survey questions derived from epidemiologic surveys (Abuse, 2019) were administered to assess ever use (yes/no) of cocaine, methamphetamine, psychedelics (e.g., LSD, acid, mushrooms), ecstasy, heroin, non-medical prescription opioid use, and non-medical benzodiazepine use. Students reporting ever use of ≥ 1 of the 7 illicit drugs during either the fall or spring of 12th grade were coded yes for illicit drug use initiation over follow-up, and the remainder were coded no (yes/no).

#### Baseline covariates

Factors considered as possible confounders of the association were included as a priori covariates literature could confound associations and therefore were included (Bachman et al. 2011; Chuang et al. 2017; Evans-Polce et al. 2014; Kilpatrick et al. 2000; Leventhal et al. 2017; Monahan et al. 2014; Roche et al. 2019). The covariates are depicted in Table 1 and described below.

##### Sociodemographic and familial influences

Sociodemographic and familial covariates included age (years; continuous variable), gender (female vs. male), parental education (binary; ≥ 1 vs. 0 parents obtained college degree), and history of substance use problems in immediate family (brothers, sisters, parents, grandparents; yes/no). Race/ethnicity was examined as a 4-level covariate. Racial/ethnic groups comprising of at least 10% of the sample (Hispanic, Asian American, and non-Hispanic white) were evaluated separately. Due to low prevalence of African American, American Indian/Alaskan Native, Pacific Islander, and multiracial individuals within the sample, these racial/ethnic groups were collapsed into one category.

##### Non-substance behaviors and traits

Behavioral health, personality traits, and psychological risk factors for substance use were assessed using valid measures (Radloff 1977; King and Chassin 2007; Whiteside and Lynam 2001). The Center of Epidemiologic Studies Depression Scale (Radloff 1977) collects past-week self-report frequency of 20 depressive symptoms on 4-point scales (0 [rarely or none of the time; 0–1 day]–3 [most or all of the time; 5–7 days]); responses are summed to indicate overall depressive symptom severity (possible range: 0–60; Cronbach’s α = 0.93). As in prior work (Kelley-Quon et al. 2019), a delinquent behavior checklist was used to collect past 6-month self-report engagement in 11 different behaviors (e.g., stealing, lying to parents), each rated from 1 (never) to 6 (10 or more times); responses summed to indicate overall severity (possible range: 11–66; Cronbach’s α = 0.64). The UPPS-P Impulsive Behavior Scale sensation-seeking subscale (assessed in spring of 9th grade) includes 12 self-statements about propensity toward pursuit of highly stimulating and novel experiences (e.g., “I generally seek new and exciting experiences”), rated from 1 (disagree strongly) to 4 (agree strongly), with responses summed (possible range: 12–48; Cronbach’s α = 0.92).

##### Additional substance use

To address a non-specific inclination towards substance use, ever (yes/no) and past 30-day use of alcohol and 7 different tobacco products (combustible cigarettes, e-cigarettes, smokeless tobacco, cigars, cigarillos, hookah, or other product) were measured (Abuse, 2019). Responses were recoded into an ever alcohol/tobacco product use covariate (0 vs. ≥ 1 alcohol or tobacco products used) and a past 30-day alcohol/tobacco use frequency covariate, derived from the average number of days that alcohol, cigarettes, hookah, cigars, and e-cigarette products were used (possible range 0–30).

### Data analysis

The primary analysis involved a series of binary logistic regression models assessing the association between baseline cannabis use and non-cannabis illicit drug use initiation over the subsequent year, each including a single cannabis use exposure variable with standard errors adjusted for within-school data clustering. For each of the 5 cannabis product exposure variables and the total number of cannabis products used (0 [reference], 1, ≥ 2), we tested separate models adjusting for baseline sociodemographic, familial, nonsubstance behavioral, and licit substance use characteristics listed above. Participants with incomplete exposure and outcome data were excluded from the analytic sample (*n* = 128). Additional sensitivity analyses examining the specificity of associations are described below. Missing covariate data was addressed using multiple imputation with chained equations to generate 10 imputed datasets. Analyses were conducted using Stata Version 16 (StataCorp Inc., College Station, TX) and reported as adjusted odds ratios (aORs) with 95% CIs. Statistical significance was set to *p* < 0.05 (two-tailed) with Benjamini–Hochberg multiple test corrections to maintain a 0.05 study-wide false discovery rate (Benjamini and Hochberg 1995).

## Results

### Descriptive analyses

The analytic sample of illicit drug never users at baseline (*n* = 2163; mean age = 17.1 [SD = 0.4] years) was 53.9% female, racially/ethnically heterogeneous (43.5% Hispanic/ Latino, 19.4% Asian,16.5% non-Hispanic white, 4.6% Black, and 16.0% of individuals were of another race/ethnicity or were multiethnic/multiracial), and included 1019 (54.5%) students with parents with college degrees (Table 1). Baseline delinquent behavior and substance use covariates were positively associated with follow-up illicit drug use initiation.

The prevalence of baseline ever cannabis use for each product is presented in Table 2; estimates varied by product: smoking flower (*n* = 558 [25.8%]), edibles (*n* = 378 [17.5%]), vaporized (*n* = 182 [8.4%]), concentrates (*n* = 84 [3.9%]), and blunts (*n* = 393 [18.2%]). At baseline, 178 (8.2%) students reported using only one cannabis product and 471 (21.8%) reported ever using ≥ 2 cannabis products. Among ever users of cannabis in any form (*n* = 649), 91 (14.0%) students used only alternative products and had never smoked cannabis flower. Descriptive statistics on past 30-day use status and frequency for each cannabis product are reported in Table 2.

Over the 12-month follow-up, 96 (4.4%) adolescents initiated the use of ≥ 1 illicit drugs; among those, 63 (65.6%) initiated the use of 1 illicit drug and 33 (34.4%) initiated the use of multiple illicit drugs over 12-month follow-up. Use initiation frequencies by follow-up broken down by specific non-cannabis illicit drugs are depicted in Table 3.

### Primary analysis

For every cannabis product, baseline ever vs. never use was associated with greater odds of initiating illicit drug use over follow-up with or without adjustment for baseline covariates (see unadjusted risk differences and aORs, respectively; Table 4). The association of baseline cannabis use with illicit drug use initiation was largest for concentrates (aOR [95% CI] = 5.88 [3.20–10.80]), followed by edible (aOR [95% CI] = 3.43 [2.32–5.08]), vaporized (aOR [95% CI] = 3.11 [2.41–4.01]), blunts (aOR [95% CI] = 2.66 [1.60–4.41], and smoked flower (aOR [95% CI] = 2.57 [1.64–4.02]) use. Relative to baseline never users of any cannabis product, baseline single cannabis product (aOR [95% CI]: 2.34 [1.26–4.34]) and poly-product (aOR [95% CI] = 3.82 [2.73–5.35]) users exhibited higher odds of illicit drug use initiation over follow-up after covariate adjustment (Table 4).

### Sensitivity analysis of specificity of associations across cannabis products

To distinguish the specificity of associations of use of each of the 5 cannabis products with follow-up illicit drug use from a non-specific involvement in use of cannabis in any form, one additional overall cannabis use involvement covariate was added to regression models—along with 11 other covariates—and retested. The cannabis use involvement covariate was derived by summing the total number of days used in the past 30 days across all methods of cannabis administration (possible range of 0–150 truncated to 0–30 for analyses). These models found that associations with follow-up illicit drug use were attenuated but remained significant for each cannabis product ever use variable and for the smoked flower, blunt, and concentrate the past 30-day exposure variables (eTable 4).

## Discussion

This study provides new evidence that the association of cannabis use with subsequent initiation of use of non-cannabis illicit drugs (cocaine, methamphetamine, psychedelics, ecstasy, heroin, non-medical prescription opioid, and/or non-medical benzodiazepine use) in adolescence extends to alternative cannabis products and poly-cannabis use. These findings advance previous longitudinal research on cannabis use and illicit drug use initiation that focused on single product exposure to smoking cannabis flower, conducted over a decade ago before alternative cannabis products became widely available.

The current findings provide updated evidence on the transition from cannabis use to other illicit drug use in the context of a recently evolved adolescent substance use landscape. Over time, cannabis is being perceived as less harmful (Sarvet et al. 2018), increasingly becoming the first substance used in the sequence of adolescent drug use (Keyes et al. 2019), and is being used in alternative forms (Knapp et al. 2018) among US adolescents. Edible and vaporized cannabis have characteristics not found in traditional smoked cannabis (e.g., availability of flavorings, appealing advertising, absence of odor, and lack of airway irritation from smoke), which might draw in adolescents who otherwise would not have used cannabis (Friese et al. 2016; Kenne et al. 2017). This trend may have been observed in the current sample, as 14.0% of cannabis ever users had never smoked cannabis flower. Additionally, the emergent opioid epidemic and unprecedented increase in US adolescent drug overdoses this decade (Curtin et al. 2017; Hasegawa et al. 2014) indicate a potential shift in the types of illicit drug use that may follow cannabis use, also observed here. In this study, non-medical prescription opioid use was the most common drug used among teens who initiated use of an illicit drug over follow-up, reinforcing concern over the possible implications of the changing cannabis landscape on pediatric health. A considerable portion of our sample *exclusively* initiated non-medical prescription drugs (e.g., painkillers, tranquilizers, and/or sedatives; 49 of 96 individuals) at follow-up (see eTable 5). As such, there is a possibility that prescription drug misuse is driving the primary findings. However, given small cell sizes, we were unable to examine the unique impact of specific cannabis product use on exclusive prescription drug use initiation.

By providing association estimates for 5 different products and distinguishing between single and poly-product cannabis use, this study confirms the presence of associations with illicit drug use initiation following all forms of alternative cannabis product use and poly-use. The difference in illicit drug use prevalence over 12-month follow-up was larger for the baseline cannabis concentrate use vs. nonuse contrast than corresponding contrasts for other cannabis products. It should be noted that cannabis concentrates were the least prevalent cannabis product used within our sample (3.9% ever used, 1.7% currently used). Low base rates likely contributed to the wide confidence intervals observed in the association between concentrate use and illicit drug use initiation. Nonetheless, only one other study on illicit drug use in youth concentrate users was cross-sectional and found that adolescent cannabis concentrate users were more likely than users of other cannabis products to have also used other illicit drugs in their lifetime (Kelley-Quon et al. 2019).

Due to the limited number of participants in our sample who were single cannabis product users, we were unable to restrict our analyses to those who exclusively used each specific cannabis product and no others. Multi-product use is important to consider when interpreting these findings; however, models examining the total number of cannabis products ever used demonstrate that both single- and polycannabis users exhibit higher odds of illicit drug use initiation over follow-up. Future research should aim to disentangle the effects of single product use in larger samples of single cannabis product users.

Although this study does not directly address the mechanisms underlying the cannabis illicit drug use association, the pattern of findings and extant literature offers clues as to why cannabis concentrate and poly-product use might be robustly associated with using other illicit drugs. To address confounders, we statistically adjusted for various sociodemographic, familial, behavioral/psychological, and substance use characteristics that may be potential confounders. Each covariate-adjusted association was statistically significant, and the aORs for cannabis concentrate exposure were substantial. While confounding explanations cannot be ruled out, it is plausible that cannabis concentrate and poly-product use could be robust risk factors for uptake of other illicit drugs. One reason is that youth who enjoy THC’s mood-altering effects may be more inclined to try other illicit drugs capable of producing pleasant psychoactive effects (Fergusson et al. 2006). Cannabis products that deliver higher doses of THC or combinations of THC alongside other cannabinoids could generate more rapid, reliable, and pleasurable psychoactive effects (Ewusi Boisvert et al. 2020; Spindle et al. 2019). As such, increased risk of illicit drug use initiation could be particularly robust in cannabis concentrate and poly-product users exposed to a high level or diversity of cannabinoids. Additionally, cannabis use during adolescence, particularly high-potency cannabis (Rigucci et al. 2016), has been associated with alterations in brain structure and function (Rigucci et al. 2016; Squeglia et al. 2009), which could, in turn, impair decision making and willingness to experiment with other illicit drugs (Kandel 2002). Additionally, peer affiliations and social networks are implicated in youth drug use (Hoffman et al. 2006; Prinstein et al. 2001; Prinstein and Dodge 2008) and could play a role in the association of cannabis use with other drug use initiation. Cannabis involvement might increase adolescents’ affiliation with substance-using peers who can provide access to illicit drugs (Kandel 2002; Oetting and Beauvais 1986). Concentrate and poly-product cannabis users might be particularly immersed in illicit drug use networks and culture and, therefore, have more positive perceptions, attitudes, and expectations about use, and ultimately greater opportunity to try illicit drugs.

Alternatively, concentrate use may merely be a proxy for more recent and frequent cannabis use patterns that, regardless of the specific product, may differentiate risk of illicit drug use initiation. This explanation is unlikely because the percentage of ever users of each product that were currently using the respective product at baseline was *lower* for concentrates than other products (Table 2). Additionally, analyses of past 30-day use also found pronounced associations for cannabis concentrate exposure. Furthermore, among past 30-day users, the mean number of days using each respective product were similar, ranging from 4.1 (blunts) to 6.1 (smoke flower). Finally, adding a total cannabis exposure covariate that summed use frequency across all products to the 11 key covariates resulted in adjusted odds of illicit drug use that were still 3.1 and 4.7 times greater for cannabis concentrate users vs. non-users for ever- and past 30-day exposures, respectively (eTable 4).

This study has limitations. First, cannabis use was based on self-reports, and adolescents both under- and over-report cannabis use (Roche et al. 2019). Our self-reported data also did not include information on uncommon cannabis products such as spliffs. Second, there are no universally accepted terms for each cannabis product, which were selected based on anecdotal reports from the youth in the study’s catchment area and previous publications (Daniulaityte et al. 2015; Peters et al. 2018). Also, there is potentially overlap between the use of vaporized and concentrate products, as vaporizers can be used with either dry flower of the cannabis plant, untreated hash oil extracts, or concentrated extracts yielded from butane solvents. Third, because of low cell counts, exposure variables for each product were binary (yes/no), leaving unclear whether graded associations are observed with increasing duration and frequency of cannabis exposure, which has been previously demonstrated with cannabis (Fergusson et al. 2006) and findings generalize across specific illicit drugs and post-initiation progression to regular illicit drug use patterns. Regardless, adolescentonset illicit drug use is an important health outcome that predicts development of substance use disorders and other health problems (Chen et al. 2009; King and Chassin 2007; Slade et al. 2008). Fourth, some of the study covariates (e.g., depression symptoms, conduct problems, licit drug use) measured contemporaneously with cannabis exposure at baseline could either be confounders that precede the risk pathway or mediators that lie along causal pathway between cannabis and other illicit drug use, raising the possibility that the aORs are deflated. Fifth, it is unclear to what extent findings from this regional sample would generalize nationally because the current sample over-represents race/ethnicity minority groups relative to the overall US population. Relatedly, while the prevalence of the use of some drugs in the current sample is similar to those in probability-based national samples, such as monitoring the future, there is not a direct concordance between our sample and national estimates which may reflect regional differences in drug use. Finally, given the observational study design, conclusions regarding whether these associations are causal cannot be made.

## Conclusion

In this prospective cohort study of Los Angeles area high school students that had never used illicit drugs by 11th grade, odds of subsequently initiating use of other illicit drugs over a 12-month follow-up were greater for those who had used each of 5 cannabis products, especially cannabis concentrate and poly-product use. Given these findings, parents, teachers, and pediatric clinicians should be aware that adolescents may use a wide spectrum of cannabis products, and youth users of cannabis products in any form may be at elevated risk for onset of illicit drug use in adolescence. Future investigation providing a more detailed characterization of illicit drug use patterns that may be associated with cannabis product use is warranted, including the specific drugs used, the frequency of use, and risk of developing drug use disorder. Additionally, if these findings were to be replicated and determined to reflect causal associations, it could be inferred that concentrate use may have elevated harms relative to using other cannabis products. If such data were to be obtained in future research, prevention programs and regulatory restrictions targeting use and sales of cannabis concentrates would merit consideration in efforts to protect adolescent health.

## Supplementary Material

Supplemental

## Figures and Tables

**Table 1:** Baseline descriptive characteristics of study sample overall and stratified by illicit drug use initiation by 12-month follow-up

Variable	Overall sample (*n* = 2163)	Initiation of illicit drug use over 12-month follow-up		
		No (*n* = 2067)	Yes (*n* = 96)	*p* value for comparison
Age, y^a^	17.1 (0.4)	17.1 (0.4)	17.1 (0.4)	.91
Female gender	1158 (53.9%)	1109 (54.0%)	49 (51.0%)	.59
Parent attended college	1019 (54.5%)	974 (54.7%)	45 (50.0%)	.43
Race/ethnicity				
White	351 (16.5%)	331 (16.3%)	20 (21.5%)	.21
Black	96 (4.5%)	93 (4.6%)	3 (3.2%)	
Hispanic/Latino	924 (43.5%)	892 (44.0%)	32 (34.4%)	
Asian	411 (19.4%)	394 (19.4%)	17 (18.3%)	
Another racial/e1thnic group^b^	340 (16.0%)	319 (15.7%)	21 (22.6%)	
Family history of drug use problems^c^	267 (14.0%)	249 (13.7%)	18 (20.2%)	.11
Depressive symptoms^a,d^	15.0 (11.9)	14.9 (11.8)	15.7 (12.6)	.59
Delinquent behavior^a,e^	13.9 (3.7)	13.8 (3.5)	17.0 (6.0)	< .001^j^
Sensation seeking^a,f^	31.8 (8.7)	31.8 (8.6)	32.8 (9.7)	.30
Past 30-day total no. alcohol, nicotine, and tobacco product use days^g^	0.7 (2.8)	0.6 (2.3)	4.0 (7.5)	< .001^j^
Ever use of alcohol, nicotine, or tobacco product^h^	1181 (54.7%)	1097 (53.1%)	84 (87.5%)	< .001^j^
Past 30-day total no. cannabis use days^a,i^	0.9 (4.0)	0.7 (3.3)	6.5 (9.7)	< .001^j^

*N* of non-missing values for each variable and denominators of percentages are listed in eTable 3 in only supplement

a Reported as mean (SD)

b Another racial/ethnic group includes Native Hawaiian/Pacific Islander, American Indian/Alaskan Native, Multiracial (I cannot choose one category), and Another (write-in response). These racial/ethnic groups were not examined separately due to small cell sizes

c Adolescents were asked, “Does anyone in your immediate family (*brothers, sisters, parents, grandparents*) have a history of drug abuse problems?”

d Scores range from 0 to 60, with higher scores indicating greater severity of past-week depressive symptoms. Center for Epidemiologic Studies Depression Scale. Each symptom is rated from 0 (rarely or none of the time; 0–1 day) to 3 (most or all of the time; 5–7 days) for 20 symptoms

e Score ranges from 11 to 66, with higher scores indicating greater frequency of engaging in 11 different delinquent behaviors in past 6 months. Each behavior is rated from 1 (never) to 6 (10 or more times) for 11 behaviors

f Scores range from 12 to 48, with higher scores indicating greater tendency toward sensation seeking. UPPS-P impulsive behavior sensation seeking scale. 12 rated from 1 (disagree strongly) to 4 (agree strongly) for 12 behaviors

g Number of days in the past 30 days where alcohol, nicotine, or tobacco (combusted cigarettes, hookah, cigars, and e-cigarettes) products were used

h Lifetime use of any alcohol, nicotine, or tobacco (combustible cigarettes, e-cigarettes, smokeless tobacco, cigars, cigarillos, hookah, or other) product

i Number of days in the past 30 days where cannabis (smoke, edible, vape, dab, blunts) products were used

j Statistically significant after Benjamini Hochberg correction for multiple testing to maintain study-wise false discovery rate below .05

**Table 2:** Prevalence and frequency of baseline ever and past 30-day use of each product

	Overall sample (*N* = 2163)		Ever users of respective product	Past 30-day users of respective product
Cannabis use variable	Ever use, *n* (%)	Past 30-day use, *n* (%)	Past 30-day use, % (95% CI)	No. days used in past 30 days, mean (95% CI)
Smoked flower	558 (25.8%)	202 (9.3%)	36.2% (32.2–40.3%)	6.1 (5.1–7.2)
Blunts	393 (18.2%)	126 (5.8%)	32.1% (27.5–36.9%)	4.3 (3.4–5.3)
Concentrates	84 (3.9%)	36 (1.7%)	42.9% (32.1–54.1%)	5.6 (2.9–8.3)
Edible	378 (17.5%)	91 (4.2%)	24.1% (19.8–28.7%)	4.7 (3.3–6.0)
Vaporized	182 (8.4%)	29 (1.3%)	15.9% (10.9–22.1%)	4.5 (2.3–6.7)
No. cannabis products				
0	1514 (70.0%)	1921 (88.8%)	N/A	N/A
1 (single product)	178 (8.2%)	98 (4.5%)	N/A	N/A
≥2 (poly-product)	471 (21.8%)	144 (6.7%)	N/A	N/A

*N/A*, not applicable

a Sample size and denominator for each value in column: smoked flower (*n* = 558), blunts (*n* = 393), concentrates (*n* = 84), edible (*n* = 378), and vaporized (*n* = 182)

b Sample size and for each in column: smoked flower (*n* = 202), blunts (*n* = 126), concentrates (*n* = 36), edible (*n* = 91), and vaporized (*n* = 29)

**Table 3:** Prevalence of initiating use of non-cannabis illicit drugs at follow-up

Drug	*n* (%)
Any illicit drug use initiation amongst overall sample	96 (4.4%)
Use initiation of specific drugs amongst those who initiated any illicit drug^a^	
Cocaine	22 (22.9%)
Methamphetamine	10 (10.4%)
LSD, acid, mushrooms, or other psychedelics	18 (18.8%)
MDMA (Ecstasy or molly)	21 (21.9%)
Heroin	4 (4.2%)
Non-medical prescription opioid use	55 (57.3%)
Non-medical prescription benzodiazepine use	36 (47.5%)

a Frequencies for each drug are not mutually exclusive because some adolescents reported ever use of more than one drug at follow-up

**Table 4:** Association of baseline cannabis ever use with non-cannabis illicit drug use initiation over follow-up

	Illicit drug use initiation over follow-up	Association with illicit drug use initiation over follow-up, OR (95% CI)	
Baseline cannabis use regressor	Unadjusted risk difference, % (95% CI)	aOR (95% CI)^a^	*p* value
Smoked flower			
Never use	REF	REF	
Ever use	8.5 (5.0, 12.1)^b^	2.57 (1.64, 4.02)	< .001^b^
Blunts			
Never use	REF	REF	
Ever use	11.3 (7.7, 14.9)^b^	2.66 (1.60, 4.41)	< .001^b^
Concentrates			
Never use	REF	REF	
Ever use	15.0 (11.3, 18.6)^b^	5.74 (3.16, 10.43)	< .001^b^
Edible			
Never use	REF	REF	
Ever use	28.8 (19.1, 38.5)^b^	3.43 (2.32, 5.08)	< .001^b^
Vaporized			
Never use	REF	REF	
Ever use	10.4 (6.7, 14.1)^b^	3.11 (2.41, 4.01)	< .001^b^
No. cannabis products			
0	REF	REF	
1	3.8 (1.1, 6.4)^b^	2.34 (1.26, 4.34)	0.01^b^
≥2	10.5 (6.9, 14.0)^b^	3.82 (2.73, 5.35)	< .001^b^

Sample excludes baseline ever users of any illicit drugs (*n* = 2163)

a Models include a single cannabis product regressor variable adjusted for baseline age, parent education, race/ethnicity, parental history of drug use problems, depressive symptoms, delinquent behaviors, past 30-day nicotine and alcohol substance use composite score (sum of total number of past 30 days of use of combustible cigarettes, alcohol, hookah, cigars, nicotine vaping), ever use of any nicotine/tobacco/alcohol product, and sensation seeking measured in spring 9th grade with separate models for each variable

b Statistically significant after Benjamini Hochberg correction for multiple testing to maintain study-wise false discovery rate below .05

## Data Availability

Data are available upon request to the corresponding author AML.

## Abbreviations

THC: Tetrahydrocannabinol
aOR: Adjusted odds ratio
CI: Confidence interval
