# Supplementary material for: Associations of alternative cannabis product use and poly-use with subsequent illicit drug use initiation during adolescence
Source: Psychopharmacology (Berl). Author manuscript; Available in PMC 2024 Sep 3. (PMC10475141; doi:10.1007/s00213-023-06330-w)
Supplement: Supplemental [file NIHMS1911606-supplement-Supplemental.docx]

**Online Supplement**

**eTable 1.** Number of observations available for each study covariate in analytic sample

**eTable 2.** Baseline prevalence of ever use of specific drugs among baseline illicit drug ever users

**eTable 3.** Comparison of fall 9^th^ grade sociodemographic and substance use characteristics between cohort enrollees included vs. excluded in the analytic sample

**eTable 4.** Association of cannabis use with illicit drug use additionally adjusted for past 30-day total cannabis involvement covariate

**eTable 5.** Frequency of exclusive initiation of specific illicit drugs at follow-up, crossed with lifetime use of each specific cannabis product.

**eTable 1. Number of observations available for each study covariate in analytic sample**

| **Variable** | **Total Sample** | **Initiation of drug use at 12-month follow-up** | |
| --- | --- | --- | --- |
|  |  | **Among those who did not initiate drug use (n=2067)** | **Among those who did initiate drug use (n=96)** |
| Age, y^a^ | 1920 | 1835 | 85 |
| Gender | 2150 | 2150 | 96 |
| Parent attended college | 1869 | 1779 | 90 |
| Race/Ethnicity | 2122 | 2029 | 93 |
| Family history of drug use problems^a^ | 1905 | 1816 | 89 |
| Depressive symptoms^b^ | 2153 | 2057 | 96 |
| Delinquent behavior^c^ | 2122 | 2029 | 93 |
| Sensation seeking^d^ | 1950 | 1862 | 88 |
| Past 30-day total no. alcohol and tobacco product use days^e^ | 2163 | 2067 | 96 |
| Ever use of alcohol or tobacco product^f^ | 2160 | 2064 | 96 |
| Note. N=2,163 Baseline never users of non-cannabis illicit drugs with complete data on cannabis exposure at baseline and non-cannabis illicit drug use follow-up.  ^a^Adolescents were asked, “Does anyone in your immediate family *(brothers, sisters, parents, grandparents)* have a history of drug abuse problems?”  ^b^Scores range from 0 to 60, with higher scores indicating greater severity of past-week depressive symptoms. Center for Epidemiologic Studies Depression Scale. Each symptom is rated from 0 (rarely or none of the time; 0-1 day) to 3 (most or all of the time; 5-7 days) for 20 symptoms.  ^c^Score ranges from 11 to 66, with higher scores indicating greater frequency of engaging in 11 different delinquent behaviors in past 6 months. Each behavior is rated from 1 (never) to 6 (10 or more times) for 11 behaviors.  ^d^Scores range from 12 to 48, with higher scores indicating greater tendency toward sensation seeking. UPPS-P Impulsive Behavior sensation seeking scale. 12 rated from 1 (disagree strongly) to 4 (agree strongly) for 12 behaviors.  ^e^Number of days in the past 30 days where alcohol, nicotine, or tobacco (combusted cigarettes, hookah, cigars, and e-cigarettes) products were used.  ^f^Lifetime use of any alcohol, nicotine, or tobacco (combustible cigarettes, e-cigarettes, smokeless tobacco, cigars, cigarillos, hookah, or other) product. | | | |

**eTable 2. Baseline prevalence of ever use of specific drugs among baseline non-cannabis illicit drug ever users**

| **Drug** | **n (%) ever use at baseline** |
| --- | --- |
| Cocaine | 198 (27.0%) |
| Methamphetamine | 123 (16.8%) |
| LSD, acid, mushrooms, or other psychedelics | 190 (26.0%) |
| MDMA (Ecstasy or molly) | 203 (27.7%) |
| Heroin | 74 (10.1%) |
| Nonmedical prescription opioid use | 535 (73.1%) |
| Nonmedical prescription benzodiazepine use | 331 (45.2%) |
| Note. Denominator for all percentages is n=732. Percentages do not sum to 100 because some adolescents reported ever use of more than one drug at baseline. | |

**eTable 3. Comparison of fall 9^th^ grade sociodemographic and substance use characteristics between cohort enrollees included vs. excluded in the analytic sample**

| **Variable** | **Analytic Sample of Baseline Illicit Drug Never Users (N=2163)** | **Excluded in Analytic Sample** | | |  |
| --- | --- | --- | --- | --- | --- |
|  |  | **Baseline Cannabis or Illicit Drug Use Data Not Available (N=395)** | **Baseline Illicit Drug Users**  **(N=732)** | **Follow-Up Illicit Drug Use Data Not Available**  **(N=106)** | **P-Value for Ombibus Comparison of 4 groups^b^** |
| Age in fall 2013, y^a^ | 14.55 (0.38) | 14.66 (0.46) | 14.60 (0.42) | 14.64 (0.49) | <.001 |
| Female gender | 1158 (53.9%) | 173 (44.6%) | 425 (58.5%) | 45 (42.9%) | <.001 |
| Parent attended college | 1019 (54.5%) | 139 (42.8%) | 289 (44.6%) | 35 (39.3%) | <.001 |
| Race/ethnicity |  |  |  |  |  |
| White | 351 (16.5%) | 46 (12.1%) | 114 (16.1%) | 9 (8.7%) | <.001 |
| Black | 96 (4.5%) | 31 (8.2%) | 33 (4.7%) | 6 (5.8%) |  |
| Hispanic/Latino | 924 (43.5%) | 200 (52.8%) | 375 (53.0%) | 58 (56.3%) |  |
| Asian | 411 (19.4%) | 41 (10.8%) | 73 (10.3%) | 10 (9.7%) |  |
| Other | 340 (16.0%) | 61 (16.1%) | 112 (15.8%) | 20 (19.4%) |  |
| Ever use of alcohol, nicotine or tobacco in fall 9^th^ grade | 585 (27.4%) | 210 (54.3%) | 475 (65.8%) | 42 (40.8%) | <.001 |
| Note. Sum of counts may not equal total sample size due to missing data. Values are n(%) unless otherwise specified.  ^a^Reported as mean (SD).  ^b^All pairwise group differences between respective group and those in analytic sample on respective outcome are statistically significant at p<.05. | | | | | |

**eTable 4. Association of cannabis use with illicit drug use additionally adjusted for past 30-day total cannabis involvement covariate**

| **Baseline Cannabis Use Regressor** | **aOR (95% CI)** |
| --- | --- |
| **Ever use (yes vs. no)** |  |
| Smoked flower | 1.85 (1.08,3.18) |
| Blunts | 1.80 (1.13,2.89) |
| Concentrates | 3.10 (1.57,6.12) |
| Edible | 2.49 (1.65,3.75) |
| Vaporized | 1.97 (1.23,3.13) |
| **Past 30 day use (yes vs. no)** |  |
| Smoked flower | 2.42 (1.22, 4.80) |
| Blunts | 3.04 (1.68, 5.48) |
| Concentrates | 4.68 (2.92, 7.52) |
| Edible | 1.86 (0.83, 4.17) |
| Vaporized | 1.13 (0.45, 2.83) |
| Note. Model includes a single cannabis product regressor variable adjusted for baseline age, parent education, race/ethnicity, parental history of drug use problems, depressive symptoms, delinquent behaviors, past 30-day nicotine and alcohol substance use composite score (sum of total number of past 30 days of use of combustible cigarettes, alcohol, hookah, cigars, nicotine vaping), ever use of any nicotine/tobacco/alcohol product, and sensation seeking measured in spring 9^th^ grade, and baseline past 30-day cannabis use composite score (sum of total number of past 30 days of use of smoking cannabis, vaping cannabis, edibles, blunt use, and concentrates). Separate models for each variable. | |

| **eTable 5.** Frequency of exclusive initiation of specific illicit drugs at follow-up, crossed with lifetime use of each specific cannabis product. | | | | | | |
| --- | --- | --- | --- | --- | --- | --- |
|  | Frequency / Total Sample | Smoked Flower | Blunts | Concentrates | Edibles | Vaporized |
| **Exclusive initiation of each specific illicit drug at follow-up** |  |  |  |  |  |  |
| Cocaine | 5 / 96 | 5 / 5 | 4 / 5 | 3 / 5 | 4 / 5 | 2 / 5 |
| Methamphetamine | 3 / 96 | 1 / 3 | 0 / 3 | 1 / 3 | 0 / 3 | 1 / 3 |
| LSD | 9 / 96 | 7 / 9 | 7 / 9 | 3 / 9 | 7 / 9 | 4 / 9 |
| Ecstasy or molly | 4 / 96 | 2 / 4 | 2 / 4 | 0 / 4 | 2 / 4 | 0 / 4 |
| Heroin | 1 / 96 | 0 / 1 | 0 / 1 | 1 / 1 | 0 / 1 | 0 / 1 |
| Prescription Painkillers | 28 / 96 | 13 / 28 | 13 / 28 | 6 / 28 | 12 / 28 | 7 / 28 |
| Tranquilizers/Sedatives | 11 / 96 | 6 / 11 | 5 / 11 | 1 / 11 | 5 / 11 | 1 / 11 |
|  |  |  |  |  |  |  |
| ANY non-medical prescription drug initiation only^a^ | 47 / 96 | 23 / 47 | 20 / 47 | 9 / 47 | 21 / 47 | 11 / 47 |
| ANY non-prescription illicit drug initiation only^b^ | 24 / 96 | 17 / 24 | 15 / 24 | 10 / 24 | 15 / 24 | 9 / 24 |
| Note.  ^a^Initiation of any non-medical prescription drug use, including prescription painkillers and/or tranquilizers/sedatives only.  ^b^Initiation of non-prescription illicit drug use, including cocaine, methamphetamine, LSD, ecstasy or molly, and/or heroin only. | | | | | | |
